# Humoral responses to SARS-CoV-2 by healthy and sick dogs during the COVID-19 pandemic in Spain

**DOI:** 10.1186/s13567-021-00897-y

**Published:** 2021-02-15

**Authors:** Ana Judith Perisé-Barrios, Beatriz Davinia Tomeo-Martín, Pablo Gómez-Ochoa, Pablo Delgado-Bonet, Pedro Plaza, Paula Palau-Concejo, Jorge González, Gustavo Ortiz-Díez, Antonio Meléndez-Lazo, Michaela Gentil, Javier García-Castro, Alicia Barbero-Fernández

**Affiliations:** 1 Biomedical Research Unit, Universidad Alfonso X El Sabio, Avda Universidad 1, 28691 Villanueva de la Cañada, Madrid Spain; 2 Vetcorner, Calle Mosén José Bosqued 2, 50012 Zaragoza, Spain; 3 ERVET-DIEZ BRU, Calle Tordesillas 4, 28925 Alcorcón, Madrid Spain; 4 Micros Veterinaria S.L., Calle Profesor Pedro Cármenes, 24007 León, Spain; 5 Faculty of Veterinary, Universidad Alfonso X El Sabio, Avda Universidad 1, 28691 Villanueva de la Cañada, Madrid Spain; 6 Laboklin GmbH & Co. KG, Steubenstraße 4, 97688 Bad Kissingen, Germany; 7 Cellular Biotechnology Unit, Instituto de Salud Carlos III, Ctra Majadahonda-Pozuelo Km 2, 28220 Majadahonda, Madrid Spain

**Keywords:** SARS-CoV-2, Dogs, Mycoplasma, Antibody, Pneumonia

## Abstract

COVID-19 is a zoonotic disease caused by SARS-CoV-2. Infections of animals with SARS-CoV-2 have recently been reported, and an increase of severe lung pathologies in domestic dogs has also been detected by veterinarians in Spain. Therefore, further descriptions of the pathological processes in those animals that show symptoms similar to those described in humans affected by COVID-19 would be highly valuable. The potential for companion animals to contribute to the continued transmission and community spread of this known human-to-human disease is an urgent issue to be considered. Forty animals with pulmonary pathologies were studied by chest X-ray, ultrasound analysis, and computed tomography. Nasopharyngeal and rectal swabs were analyzed to detect canine pathogens, including SARS-CoV-2. An additional twenty healthy dogs living in SARS-CoV-2-positive households were included. Immunoglobulin detection by several immunoassays was performed. Our findings show that sick dogs presented severe alveolar or interstitial patterns with pulmonary opacity, parenchymal abnormalities, and bilateral lesions. The forty sick dogs were negative for SARS-CoV-2 but *Mycoplasma* spp. was detected in 26 of 33 dogs. Five healthy and one pathological dog presented IgG against SARS-CoV-2. Here we report that despite detecting dogs with α-SARS-CoV-2 IgG, we never obtained a positive RT-qPCR for SARS-SoV-2, not even in dogs with severe pulmonary disease; suggesting that even in the case of canine infection, transmission would be unlikely. Moreover, dogs living in COVID-19-positive households could have been more highly exposed to infection with SARS-CoV-2.

## Introduction

The year 2020 has seen an international health emergency generated by the emerging zoonotic coronavirus SARS-CoV-2, which began its expansion in the end of the 2019 in Wuhan (China) and caused a pandemic within a few months [1, 2]. SARS-CoV-2 infection can lead to COVID-19, a pathology with various clinical manifestations, and in its severest form is mainly associated with lung injury and pathology similar to macrophage activation syndrome, such as hyperinflammation and lung damage by an uncontrolled activation and proliferation of T lymphocytes and macrophages [3, 4].

Four genera of coronaviruses have been described: Alphacoronavirus, Betacoronavirus, Gammacoronavirus and Deltacoronavirus (α-CoV, β-CoV, γ-CoV and δ-CoV) according to their genetic structure. α-CoV and β-CoV infect mammals, and in addition to humans, have been also described in dogs and cats. Mostly, they are responsible for respiratory infections in humans and gastroenteritis in animals. In dogs, canine enteric coronavirus (CCoV), an α-CoV, causes an enteritis of variable severity that is rarely fatal but leads to the development of immunity. However, some recovered dogs become carriers with the ability to infect other dogs. However, canine respiratory coronavirus (CRCoV), which belongs to the β-CoV (like SARS-CoV-2), causes respiratory symptoms in dogs, with generally mild clinical signs [5] and occasionally as a coinfection with other respiratory pathogens. Recently, the first cases of asymptomatic dogs infected with SARS-CoV-2 have been described [6].

Due to the zoonotic origin of SARS-CoV-2 and the described transmission between species, a hypothesis of the transmission and spread among animals of different species has become more plausible [7]. Cases of infected cats, dogs, tigers, lions, minks and ferrets have been reported during SARS-CoV-2 outbreaks, all of which had had close contact with infected people [6, 8]. Some data suggest that transmission from minks to humans, cats and dogs has occurred on farms.

The World Organization for Animal Health (OIE) stated that some animals can become infected by being in permanent contact with infected people, although they note that there is no evidence pointing to a role of infected pets in the spread of SARS-CoV-2. To date, no cases of transmission from domestic animals to humans have been described [9]. Some researchers have reported that dogs whose owners were positive for SARS-CoV-2 showed negative serological results to SARS-CoV-2, postulating that pets are not virus carriers [10]. By contrast, there have been some cases of companion dogs reported to be positive by quantitative reverse transcription PCR (RT-qPCR) detection [6] and others that have developed neutralizing antibodies against SARS-CoV-2 [11]. RT-qPCR-positive dogs have been detected worldwide (Hong Kong, Denmark and USA, but none in Spain), all of them in close contact with SARS-CoV-2 positive humans [9]. Currently, 28 positive dogs to SARS-CoV-2 have been reported by RT-qPCR around the world. Less than half of them were asymptomatic [6, 12]. One presented mild respiratory illness, and only one presented also neutralizing antibodies accompanied with hemolytic anemia [13]. On the other hand, two PCR-negative dogs have developed neutralizing antibodies. One was asymptomatic and the other had breathing problems, but it is not clear if this was related to the infection [13]. Further, molecular testing of 3500 companion dogs, cats and horses were done by IDEXX Company in USA and Korea and no positive cases were found [14]. No positive cases have been reported in dogs exposed to SARS-CoV-2 in France [15]. Another recent study in Italy carried out with pets has shown that none of the 817 animals studied were positive for SARS-CoV-2 by RT-qPCR test but 13 dogs and 6 cats had neutralizing antibodies [11].

In this report, we show that during the spring months of the year 2020, coinciding with the pandemic, an increase of severe lung pathologies in dogs was detected by veterinarians in Spain. Therefore, it is important to determine the infectious agent(s) and a potential role of SARS-CoV-2 infection. It is necessary also to describe the pathological processes that could occur in those animals, which could be infected by SARS-CoV-2 and which show symptoms similar to humans affected by COVID-19. It is also highly relevant to determine if dogs could become infected in a home environment where close human-pet interactions occur. Here, we describe the study of sick and healthy dogs regarding potential infection with SARS-CoV-2.

## Materials and methods

### Clinical study

A prospective study with forty dogs presenting pneumonia was performed between April and June 2020 in Spain. This study was conducted in several hospitals and clinics, in Madrid and Zaragoza (Spain). The inclusion criteria were to present at least three of the following clinical signs: fever (rectal temperature higher than 39.5 °C), cough, fatigue, tachypnoea (higher than 30 breaths per min), or crackles on lung auscultation. Gastrointestinal signs (vomiting and/or diarrhea) and tachycardia (higher than 130 beats per min) were also recorded. Dogs without evidence of pneumonia on imaging tests and dogs presenting signs that suggested cardiogenic oedema or tumors were excluded. The clinical diagnosis of pneumonia was through various imaging tests, including thoracic ultrasound. For a complete work-up dorsoventral and laterolateral thoracic radiographs, abdominal ultrasound, and complete hematologic work-up were performed on all dogs that had owner authorization. A clinical follow-up of all patients was performed and mortality was also recorded.

Twenty healthy dogs living with people with a SARS-CoV-2-confirmed infection were included as animals exposed to the virus. These dogs did not present any symptoms at the time of sample taking, and therefore were considered healthy animals. The inclusion criterion was the animals lived in homes where at least one person had been diagnosed with COVID-19. Exclusion criteria were pregnant females and dogs diagnosed with any ongoing pathology or infection.

The study was approved by the Ethical Committee of the Faculty of Health Sciences, Alfonso X el Sabio University and all dog owners gave written informed consent.

### Image analysis

**Figure 1:**
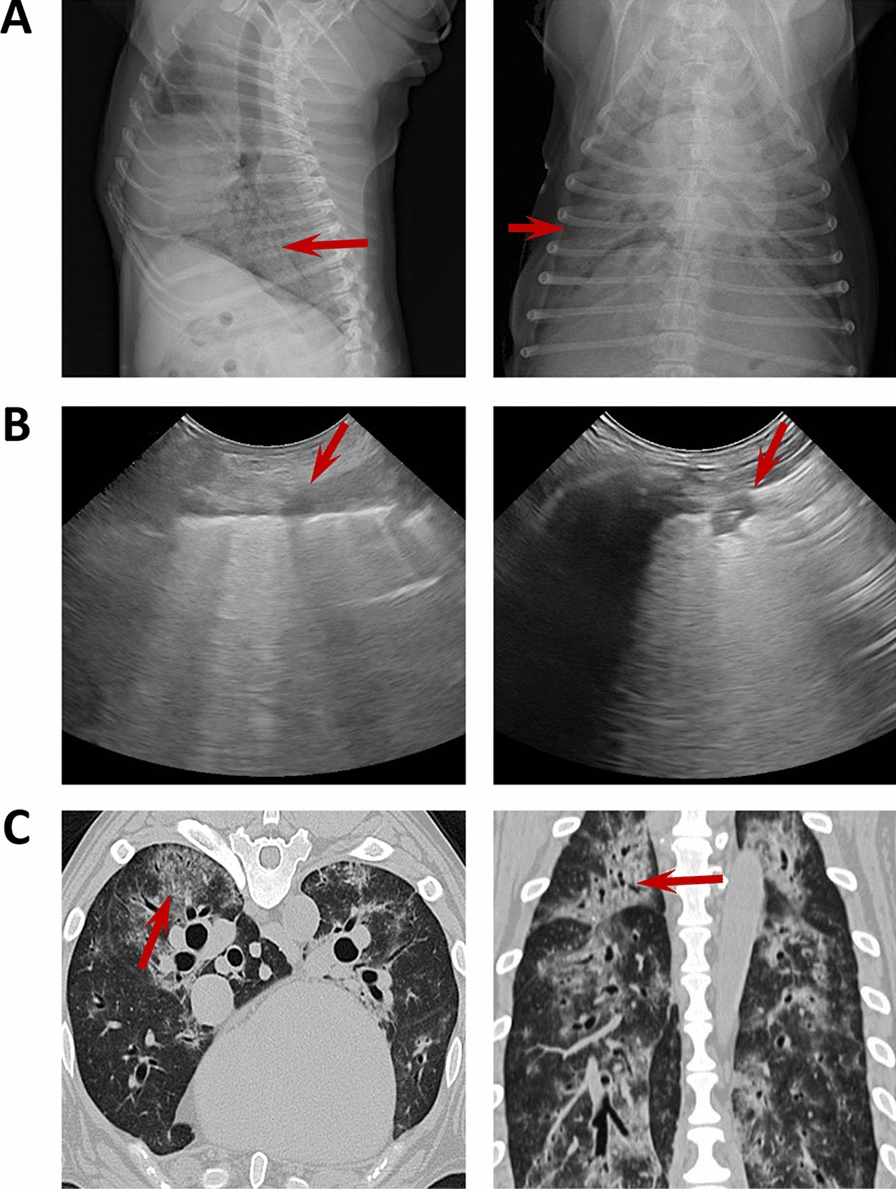
**Imaging with chest radiograph, sonographic images and CT of three different dogs.** **A** Thoracic radiograph made in right lateral (left) and dorsoventral (right) showing a generalized severe interstitial opacity accentuated in the caudodorsal (arrows). **B** Sonographic images of two patients with severe dyspnea showing a diffused B line (left; arrow) and consolidation focal lesions (right; arrow). **C** Transverse (left) chest CT images showing bilateral focal peripheral ground-glass opacities with intralobular and interlobular smooth septal thickening (arrow); sagittal (right) chest CT images showing diffuse opacities with consolidation and bronchial wall thickening (arrow).

Chest X-ray (CXR) was performed in two projections (Right Laterolateral (LL)/Dorsoventral (DV)). Thoracic radiographs and a study ultrasound were performed in sick dogs using a portable ultrasonographic device (My Lab Alpha Vet Esaote S.P.A, Barcelona, Spain) equipped with two multifrequency linear (3–13 MHz) and microconvex (8–12 MHz) transducer. The Vet BLUE protocol was used to study the lungs of each patient, the images were acquired at 4 acoustic windows on each side of the thorax at standardized anatomic sites (caudal, perihilar, middle and cranial), providing a total of 8 sites/patient. The pathological lung was recognized when the ultrasound lung rockets also called B lines were observed (Figure 1) or by the presence of other pulmonary ultrasound findings for consolidation (crushing, tissue, nodule sign) (Figure 1).

Computed tomographic (CT) scans were obtained using 64-multidetector scanners (Aquilion, Toshiba) with all dogs positioned in sternal recumbency under general anaesthesia, the CT scan was performed during temporary apnoea induced by hyperventilation. CT scans were examined by a radiologist. The general distribution of the lung lesions was classified as generalized, focal and uni or bilateral. The presence or absence of pleural effusion was studied with all techniques.

### Immunoglobulins detection by immunoassays

Blood samples were collected in BD vacutainer plasma separator tubes (BD PST II, Becton Dickinson, Franklin Lakes, NJ, USA), plasma was obtained and frozen at -20 °C until analysis. As a negative control, samples from dogs with no known virus exposure were used, kindly given by Centro de Transfusión Veterinario (Madrid, Spain).

To determine immunoglobulins (IgG) against SARS-CoV-2 in dog plasma samples, a highly sensitive SARS-CoV-2 Spike S1 protein ELISA Kit was used (MyBioSource, San Diego, CA, USA) following the manufacturer’s instructions. Captured IgG against SARS-CoV-2 S1 protein were detected by goat anti-dog IgG (H&L) polyclonal antibody conjugated with horse radish peroxidase (HRP) (Invitrogen, Carlsbad, CA, USA). Absorbance was measured at 450 nm using the Varioskan LUX, ver. 1.00.37 (Thermo Fisher Scientific, Waltham, Massachusetts, USA). Results were calculated using the SkanIt Software 5.0 for Microplate Readers RE, ver. 5.0.0.42 (Thermo Fisher Scientific). Values > 2.5OD of the negative control were considered as positives, with 2.14OD being the cutoff value to determine IgG against SARS-CoV-2.

To determine antibodies (IgM and IgG) against canine coronavirus that causes infections in enteric tract (CCoV), dog plasma samples were analyzed by two different commercial enzyme immunoassays (Eurovet veterinaria S.L., Daganzo de Arriba, Madrid, Spain) following the manufacturer’s instructions. Both immunoassays recognize two different strains of CCoV, CCoV type I (CCoV-I) and CCoV type II (CCoV-II). Absorbance was measured at 450 nm using the Varioskan LUX, ver. 1.00.37 (Thermo Fisher Scientific). Results were calculated using the SkanIt Software 5.0 for Microplate Readers RE, ver. 5.0.0.42 (Thermo Fisher Scientific). The S/P ratio was calculated as (ODsample—MVODNC)/(MVODPC—MVODNC), where MV was mean value, NC was negative control and PC was positive control. When quantifying IgM, samples with the S/P ratio ≥ 0.250 were considered positives. When quantifying IgG, samples with the S/P ratio ≥ 0.240 were considered positives.

To determine neutralizing antibodies (IgG) against canine adenovirus (CAV), canine parvovirus (CPV) and canine distemper virus (CDV), plasma samples were analyzed by an ELISA in solid phase (ImmunoComb Canine VacciCheck, Biogal Galed Laboratories Acs., Galed, Israel). The assays were performed following the manufacturer’s instructions. ImmunoComb images were digitalized and spot densities were quantified using Image Lab™ 5.0 Software (Bio-Rad, Hercules, CA, USA). Arbitrary units were calculated as follows: (sample spot intensity—sample mean background intensity)—(positive reference spot intensity—positive reference spot mean background intensity), and negative/positive criterion was applied following manufacturer’s instructions.

### PCR analysis

Nasopharyngeal swabs (all dogs) and rectal swabs (sick dogs only) were collected and analyzed by Laboklin GmbH & Co. (Bad Kissingen, Germany) using conventional PCR or real-time PCR (qPCR and RT-qPCR). Swabs were incubated in 750 µL MagNA Pure DNA Tissue Lysis Buffer (Roche Diagnostics GmbH, DE-Mannheim, Germany) plus 75 µL Proteinase K (Carl Roth GmbH + Co. KG, DE-Karlsruhe, Germany) for 1 h at 65 °C. Automated isolation of nucleic acids (RNA and DNA) was performed with the MagNA Pure 96 system from Roche Diagnostics GmbH according to manufacturer's instructions. Nasopharyngeal swabs from sick dogs were tested for canine adenovirus type 2 (CAV-2) [16], *Bordetella bronchiseptica* [17], CDV [18], canine parainfluenza virus (CPIV) (in-house method), canine influenza A virus (CIV) [19] and canine herpesvirus-1 (Canid alphaherpesvirus-1: CaHV-1) [20] by Taqman real-time PCR on a LightCycler®96 (Roche Diagnostics, Basel, Switzerland) and for *Mycoplasma* spp. [21] by conventional PCR. Swabs from all sick dogs, and swabs from healthy dogs that presented α-SARS-CoV-2 IgG, were also tested for SARS-CoV-2 [22] by Taqman real-time PCR on a LightCycler®96 (Roche Diagnostics).

### Histopathology

Lungs of two dogs (SER209 and SER222) were histologically evaluated after necropsy. The macroscopic exam evaluated congestion, oedema and the lung injury pattern, evaluating whether there is scattered involvement by areas or if it is generalized throughout the lungs, as well as the presence and type of lesions. Lung samples were fixed in formalin 4% for 24 h, paraffin-embedded and 3 µm thick sections were obtained and stained with hematoxylin–eosin.

### Statistical methods

Categorical variables were presented as percentages. For continuous variables, data distribution normality was evaluated with the Kolmogorov–Smirnov test. Continuous data were presented as mean with standard deviation (SD) or median with interquartile range (IQR).

## Results

Forty pathologic dogs met the inclusion criteria with the mean age of 8 years (range: 2 months to 13 years). Fifteen breeds were recorded with the most common being Cross-Breed 22.5% (9/40), Yorkshire terrier 12.5% (5/40), and German shepherd 10% (4/40). There were 22 females and 18 males.

The most common clinical signs were crackles on lung auscultation (100%), followed by cough (92.5%), tachypnoea (80%), fatigue (72.5%), tachycardia (60%), fever (57.5%), vomiting (37.5%) and diarrhoea (35%) (Table 1). The radiographic findings in the 40 analyzed dogs were consistent with mild to severe alveolar or interstitial pattern, with pulmonary opacity accentuated in the caudodorsal lung field. In 32.5% (13/40) of dogs a generalized increase in pulmonary opacity affecting all lung lobes was noted (Figure 1A). A focal alveolar infiltrate in the cranioventral lung field was detected in 50% (20/40) of dogs. One or more pathological findings were observed during the ultrasound examination of the patients. The main sonographic features were dispersed B-line and rocket sign (100%; 31/31), partially diffused B-line (80.65%; 25/31) and pulmonary consolidations focal lesions (48.39%; 15/31). Complications such as pleural effusion were rarely seen (4%; 1/25) and pneumothorax was not detected (Figure 1B). The CT findings were pulmonary parenchymal abnormalities (Figure 1C) and the lung lesions were bilateral in all evaluated dogs.

**Table 1:** Epidemiologic characteristics, clinical features, and mortality

	Frequency (*N* = 40)	Percentage (%)
Epidemiologic characteristics		
Gender		
Female	22	55.0
Male	18	45.0
Age (years)	8.0^a^ (2 months-13 years)	3.4^b^
Clinical features		
Crackles (lung auscultation)	40	100
Cough	37	92.5
Tachypnoea (> 30 breaths per minute)	32	80.0
Fatigue	29	72.5
Tachycardia (> 130 beats per minute)	24	60.0
Fever (> 39.5 °C)	23	57.5
Vomiting	15	37.5
Diarrhoea	14	35.0
Mortality	17	42.5

^a^Mean

^b^Standard deviation

In order to assess the overall status of dogs, and considering that the number of white blood cells is frequently altered in an infection, we consider relevant to perform a hematologic evaluation on twenty-four pathologic patients. The count of white blood cells (WBC) was out of normal range in 58.3% of dogs with the number of neutrophils being abnormal in 75%, lymphocytes in 37.5% and monocytes in 45.8% (Table 2).

**Table 2:** Hematologic peripheral blood analysis

Hematologic	Out of range parameters	Normal range	Mean	Standard deviation
	*N* = 24 (%)			(SD)
WBC (× 10^3^cells/µL)	14 (58.3)	6.0–17.0	20.5	9.7
Neutrophils (× 10^3^cells/µL)	18 (75)	3.0–11.5	16.7	9.1
Lymphocytes (× 10^3^cells/µL)	9 (37.5)	1.0–4.8	1.9^a^	1.0–3.3^b^
Monocytes (× 10^3^cells/µL)	11 (45.8)	0.2–1.4	1.2^a^	0.7–1.7^b^

*WBC* White Blood Cells

^a^Median

^b^Interquartile range (IQR)

Considering the altered immune cell numbers observed in peripheral blood and the clinical course, we proceeded to evaluate possible pulmonary pathogens. In order to determine whether the observed pathologies could be related to a SARS-CoV-2 infection or to other pathogens, PCR analysis was performed. All forty dogs were negative for SARS-CoV-2 (Table 3). Thirty-three dogs were additionally analyzed for a complete profile including the most common canine infectious agents. All of them were negative for CPIV, CIV, an

**Table 3:** Infectious pathogens of sick dogs

	CAV-2	*Mycoplasma* spp.	*Bordetella bronchiseptica*	CDV	CPIV	CIV	CaHV-1	SARS-CoV-2
SER 01	−	+	+	−	−	−	−	−
SER 02	−	+	−	−	−	−	−	−
SER 03	−	+	−	−	−	−	−	−
SER 04	nd	nd	nd	nd	nd	nd	nd	−
SER 05	−	+	−	−	−	−	−	−
SER 06	nd	nd	nd	nd	nd	nd	nd	−
SER 07	nd	nd	nd	nd	nd	nd	nd	−
SER 08	nd	nd	nd	nd	nd	nd	nd	−
SER 09	−	+	−	−	−	−	−	−
SER 10	−	+	−	−	−	−	−	−
SER 11	−	+	−	−	−	−	−	−
SER 12	−	+	−	−	−	−	−	−
SER 13	−	+	−	−	−	−	−	−
SER 14	−	+	−	−	−	−	−	−
SER 15	−	+	−	+	−	−	−	−
SER 16	−	+	−	−	−	−	−	−
SER 17	−	+	−	−	−	−	−	−
SER 18	−	+	−	+	−	−	−	−
SER 202	−	–	−	−	−	−	−	−
SER 204	−	+	−	−	−	−	−	−
SER 205	−	+	−	−	−	−	−	−
SER 206	−	+	−	−	−	−	−	−
SER 207	−	+	−	−	−	−	−	−
SER 208	−	+	−	−	−	−	−	−
SER 209	−	+	−	−	−	−	−	−
SER 210	−	+	−	−	−	−	−	−
SER 211	−	−	−	−	−	−	−	−
SER 212	−	−	−	−	−	−	−	−
SER 213	−	−	−	−	−	−	−	−
SER 214	+	+	–	–	−	−	−	−
SER 217	–	+	+	+	−	−	−	−
SER 220	–	+	–	+	−	−	−	−
SER 222	–	+	–	–	−	−	−	−
SER 223	–	–	–	–	−	−	−	−
SER 233	–	–	–	+	–	–	–	−
SER 234	–	+	–	+	–	–	–	−
SER 235	–	–	–	–	–	–	–	−
SER 301	nd	nd	nd	nd	nd	nd	nd	−
SER 302	nd	nd	nd	nd	nd	nd	nd	−
SER 303	nd	nd	nd	nd	nd	nd	nd	−

CAV-2: canine adenovirus type 2; CDV: canine distemper virus; CPIV: canine parainfluenza virus; CIV: canine influenza A virus; CaHV-1: canine herpesvirus-1; SARS-CoV-2: severe acute respiratory syndrome coronavirus 2. Presence (+), absence (–), not determined (nd)

d CaHV-1. *Mycoplasma* spp. and CDV were detected as a single agent in 57.6% (19/33) and 3% (1/33), respectively (Table 3).

**Figure 2:**
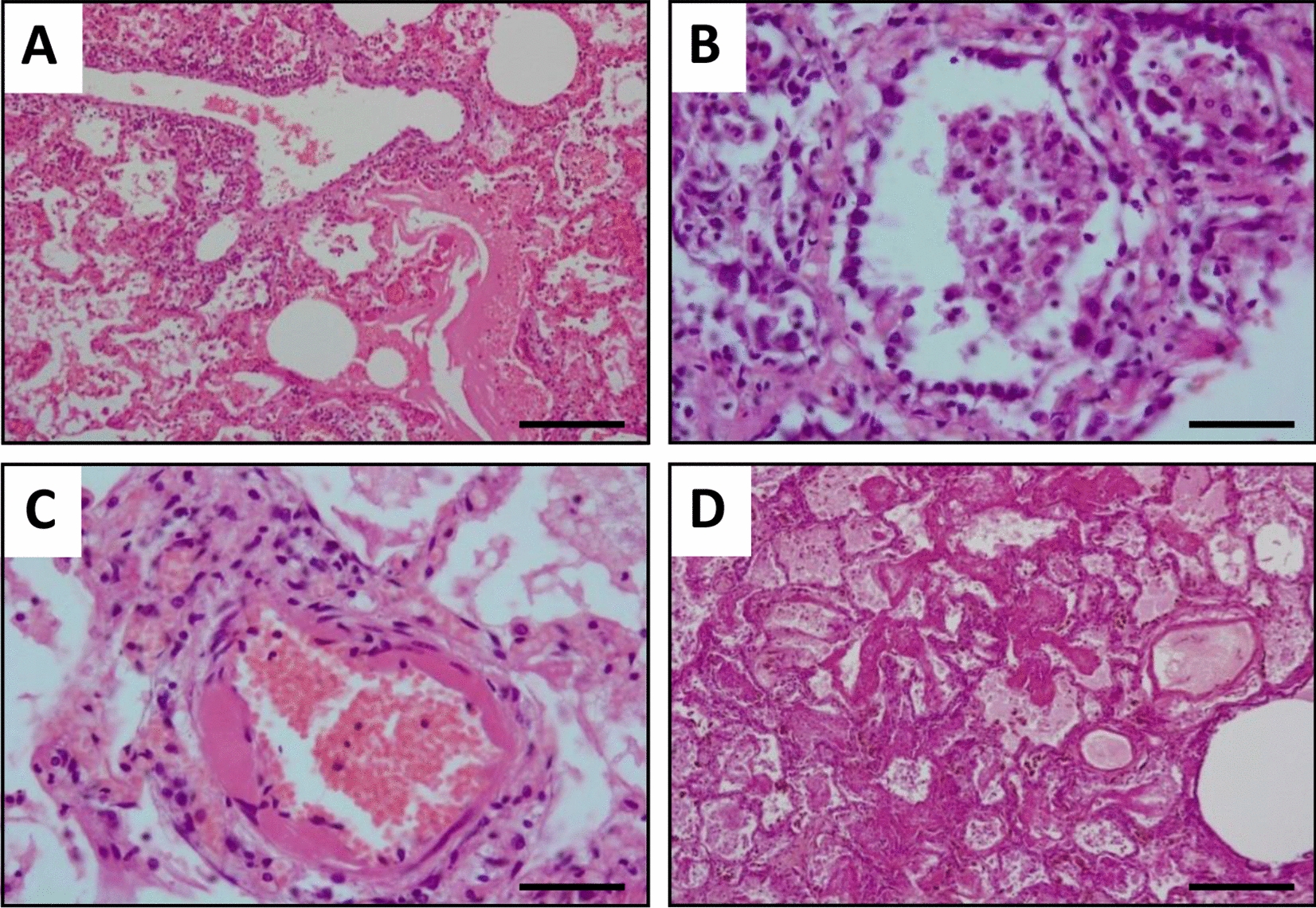
**Histopathological study of lung tissues in sick dogs.** Representative images of hematoxylin and eosin stained necropsy samples are shown. **A** Sample showing moderate vasculitis with rich-protein alveolar oedema and haemorrhages. **B** Lung tissue showing alveolar lined by type II pneumocytes and inflammatory infiltrate in the alveolar lumens. **C** Arteriolar wall hyalinosis is shown. **D** Diffuse alveolar damage with oedema and intra-alveolar hyaline membranes are shown. Scale bar: 200 µm (**A**, **D**) and 50 µm (**B**, **C**).

The pathologies in our patients were severe with 42.5% (17/40) dying of pneumonia during follow-up (Table 1), all from respiratory distress. The patients who survived had permanent radiographic changes in the lung parenchyma, with a predominance of the interstitial pattern, as well as reduced respiratory capacity. Two of the deceased dogs were necropsied in order to study lung tissue damage. The macroscopic examinations showed congested, oedematous, and consolidated lung parenchyma with patchy involvement in both dogs. Both animals presented severe interstitial pneumonia with diffuse alveolar damage. Furthermore, one dog (SER209) presented extensive congestion, haemorrhages (Figure 2A), and fibrin sheaths in the alveolar lumen with an inflammatory infiltrate of lymphocytes and macrophages (Figure 2B), with scattered syncytia. Occasionally, alveoli are lined by Type II pneumocytes (Figure 2B). There was multifocal vasculitis with periarteriolar lymphocyte infiltrate and occasional vascular wall hyalinosis (Figure 2C). Animal SER222 showed intense autolytic changes with severe acute alveolar damage, vascular lesions such as congestion, rich-protein alveolar oedema (Figure 2D), and hyaline membranes that occluded alveolar lumina. Scant and disperse inflammatory cells, mainly macrophages with intracytoplasmic brown granular pigment (compatible with hemosiderin) were observed in alveolar septa. Interestingly, some of these findings, particularly the scattered syncytia are usually present in some viral infections [23].

After testing for possible pathogens in the diagnosed dogs and analyzing the evaluated tissues, we set out to study the immune response against some of these infectious agents in 17 dogs. Further, we also decided to study 20 dogs that lived with people diagnosed with SARS-CoV-2 infection, as a group of dogs potentially exposed to the virus, and which did not present symptoms at the time of sampling. First of all, information about vaccination was gathered to determine the immune status of dogs. Ten sick dogs had been vaccinated routinely according to recommendations by veterinarians but eight sick dogs did not receive any vaccine (Additional file 1). We have not detected any association between the vaccination patterns of pathological animals compared to healthy dogs.

**Figure 3:**
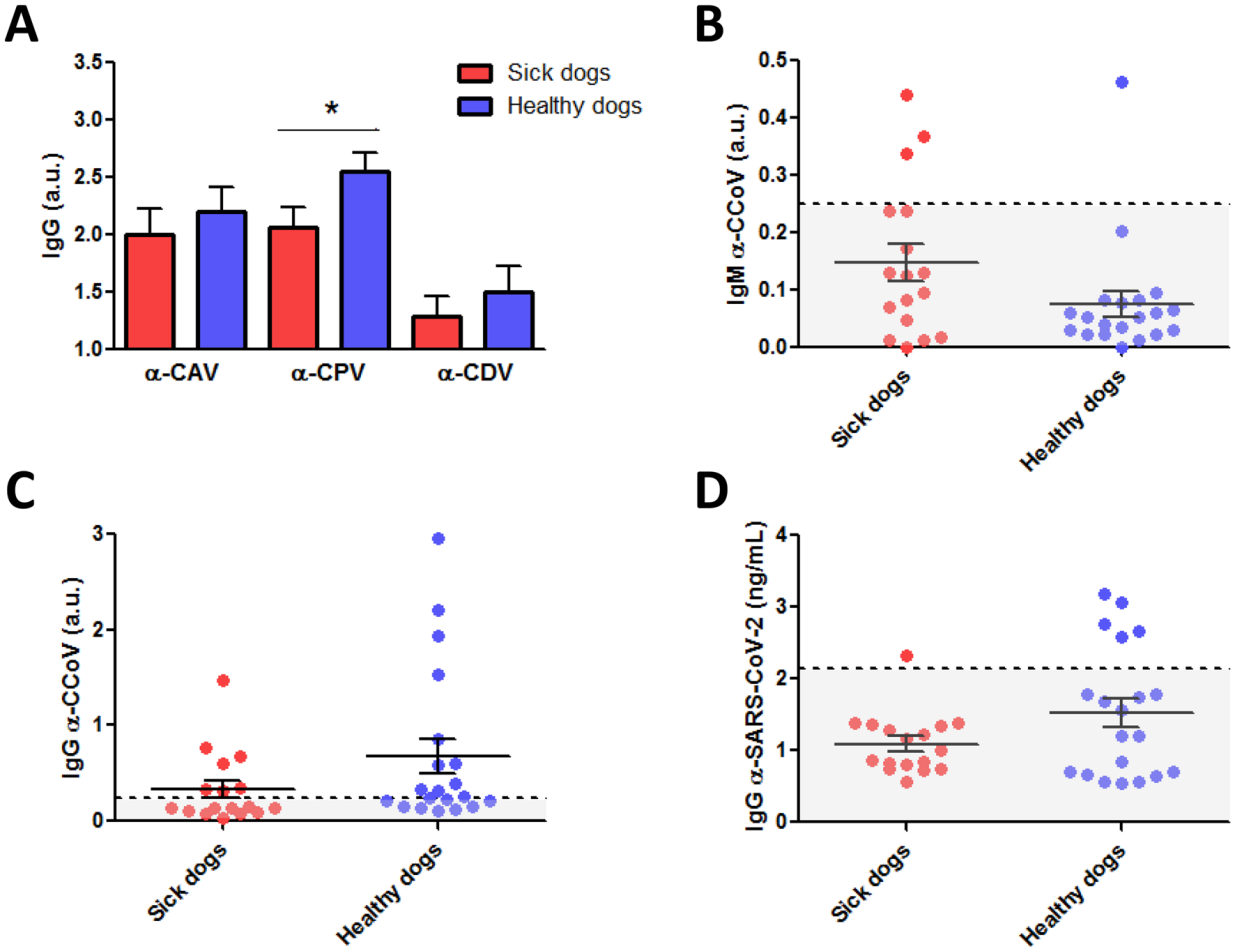
**Immune response of sick and healthy dogs. A** Quantification of immunoglobulins (Ig) G against canine adenovirus (CAV), canine parvovirus (CPV) and canine distemper virus (CDV) in sick (red bars; *n* = 17) and healthy (blue bars; *n* = 20) dogs. Mean + standard error of the mean (SEM) are shown. Mann–Whitney *U* test; *p < 0.05. Scatter dot plots showing the quantification of IgM (**B**) and IgG (**C**) against canine coronavirus (CCoV) in sick and healthy dogs. Mean ± SEM (bars) and individual values (dots) are shown. Dotted line indicates the range of positives. **D** Quantification of IgG against severe acute respiratory syndrome coronavirus 2 (SARS-CoV-2) in sick and healthy dogs. Mean ± SEM (bars) and individual values (dots) are shown. Dotted line indicates the range of positives. a.u.: arbitrary units.

Immunoglobulins G (IgG) against CAV, CPV and CDV were analyzed in peripheral blood samples from these sick and healthy dogs (Figure 3 and Additional file 2). Further, antibodies (IgM and IgG isotypes) against canine coronavirus that affects the enteric tract (CCoV), and also IgG against SARS-CoV-2 were studied for both groups (Figure 3 and Additional file 2). The number of dogs that presented IgG antibodies against SARS-CoV-2 was higher in the group of healthy dogs in COVID-19-positive households (25%; 5/20), compared to the pathological ones (5.88%; 1/17). Interestingly, the sick dog that presented antibodies against SARS-CoV-2 was negative for the detection of the virus in swabs studied by RT-qPCR, however *Mycoplasma* spp. and CDV were detected in this patient (Additional file 2 and Table 3). All five α-SARS-CoV-2 IgG-positive healthy dogs were negative to SARS-CoV-2 by RT-qPCR. All of them showed the same pattern of antibodies against some of the studied pathogens, with positives for α-CAV IgG, α-CPV IgG and α-CDV IgG (Additional file 2). Nevertheless, two of the five presented α-CCoV IgG while the remaining three were not protected against canine coronavirus. Twelve healthy dogs presented α-CCoV IgG with two of these twelve being positive for α-SARS-CoV-2 IgG (Figure 3 and Additional file 2). Seven pathological dogs presented α-CCoV IgG, but in this group all were negative for α-SARS-CoV-2 IgG (Figure 3 and Additional file 2).

## Discussion

Dogs are currently considered to be less susceptible hosts for SARS-CoV-2 than cats or minks, despite the fact that several positive RT-qPCR test results in dogs have been reported [6]. Surveillance data from IDEXX laboratories, show no positive results for SARS-CoV-2 in any of more than 1500 dog specimens submitted for respiratory PCR panels, suggesting that transmission from human to pet is very rare. However, veterinarians in Spain have observed an increase in aggressive lung pathologies in dogs during the human COVID-19 pandemic that have not responded to conventional antibiotic treatments. Moreover, the mortality rate due to respiratory disease is typically very low (1.2% and only 0.3% due to pneumonia) [24, 25]. Nevertheless, we found a mortality rate of 42.5% during follow-up. Dogs with very aggressive lung diseases showed a very similar appearance to humans presenting COVID-19 [26]. We cannot rule out the existence of an acute respiratory distress syndrome (ARDS) that generates the described pathological patterns. However, these cases in veterinary medicine are very uncommon and tend to have known etiology, usually a sequel to bacterial pneumonia, aspiration pneumonia, sepsis, or shock [27].

Historically, the most common pathogens associated with complex canine infectious respiratory disease have been CPIV, CAV-2, *Bordetella bronchiseptica*, *Streptococcus equi* subsp. *zooepidemicus*, *Mycoplasma cynos*, CaHV-1, CDV, CIV, and CRCoV [25]. In our study, we detected eight of 33 analyzed dogs presenting classical primary respiratory pathogens, and we also detected IgM for CCoV in four dogs (3/17 pathological dogs and 1/20 healthy dog). However, the presence of CRCoV is detected more frequently in dogs with mild clinical signs than in dogs with moderate or severe clinical signs [5]. Therefore, we can assume that it was not the agent responsible for the severe respiratory pathologies in these three dogs.

Interestingly, 26 of 33 analyzed dogs showed a positive test for *Mycoplasma* spp. Many *Mycoplasma* spp. are commensal organisms that colonize the mucous membranes of the respiratory tract, and their role in canine infectious respiratory disease is not clear. Moreover, *Mycoplasma cynos* is the only species of the genus *Mycoplasma* significantly associated with pneumonia in dogs but it is still also unclear if *M. cynos* is a primary or secondary pathogen in dogs, because it can be cultured from the lungs of dogs, both with and without other identifiable infectious agents [28]. In a European study of dogs with canine infectious respiratory disease, seroprevalence of *Mycoplasma* spp. levels ranged from 20.7 to 61.9% [29], but in another study with healthy dogs, mycoplasma were isolated from 78 to 93% of throat swabs [25]. Moreover, mycoplasma infections are usually associated with other infections. It is interesting to note that mycoplasma coinfections are very common in COVID-19 human patients [30], and it also has been suggested that a coinfection or activation of latent mycoplasma infection in COVID-19 disease may be highly significant in determining a fatal disease course [31, 32].

Normally, the therapy response when treating canine respiratory tract diseases with drugs (antibiotics, bronchodilators, anti-inflammatories, antitussives, decongestants, mucolytics, mucokinetics or expectorants) is adequate at alleviating the symptoms or reversing the disease. Nevertheless, our patients did not respond adequately to the therapeutic protocol. A major common pathogen has not been detected in our patients, so at the moment the causative agent of the pathologies is unknown. Further, the number of deaths was more than 30 times higher than expected without clarified etiology and curiously coincidental to a peak of the COVID-19 pandemic in Spain. When analyzing deceased dogs, interstitial pneumonia that usually courses with nonspecific lesions was detected. This manifestation has also been described in canine pathologies, such as canine distemper, herbicide poisoning (paraquat) and systemic processes (principally, septicemia or uremia) [23]. However, it should be noted that the observed lesions are similar to those described for humans with COVID-19 [2]. Especially, striking lesions observed in vessels, both lymphocytic vasculitis and the hyalinosis of the arteriolar wall [33]. However, all of them were negative for RT-qPCR tests for SARS-CoV-2 using nasopharyngeal and rectal samples. These results agree with a large-scale study that was recently carried out to assess SARS-CoV-2 infection in 817 companion animals living in northern Italy, in which similarly none of the animals tested positive using RT-qPCR [11]. Likewise, it should be considered that viral particles have been detected in the skin endothelium of human patients despite the fact that they were negative when nasopharyngeal swabs were tested by RT-qPCR. Therefore, it would be useful to analyze SARS-CoV-2 by immunohistochemistry and/or RT-qPCR in necropsy samples from our patients [34].

In a previous study on the presence of immunoglobulins against SARS-CoV-2 in peripheral blood of pets, 487 dogs were tested in China. They were serologically negative for anti-SARS-CoV-2 IgGs. Among them, sera from 15 pet dogs and 99 feral dogs were collected from Wuhan City, but it should be noted that only one pet dog living with a confirmed human COVID-19 patient presented antibodies against SARS-CoV-2 [10]. However, in the Italian study, 3.4% of 188 dogs (and 3.9% of 63 cats) had measurable SARS-CoV-2 neutralizing antibody titers. None of these animals with neutralizing antibodies displayed respiratory symptoms at the time of sampling. As expected, dogs from SARS-CoV-2-positive households seemed to be significantly more likely to test IgG positive than those from SARS-CoV-2-negative households [11]. Finally, other researchers have determined that only half of the dogs artificially inoculated with SARS-CoV-2 seroconverted [8].

Here we detected specific anti-SARS-CoV-2 canine immunoglobulins in one sick dog (1/17) and in 25% (5/20) of dogs living in COVID-19-positive households, indicating their susceptibility to SARS-CoV-2 infection. Seropositivity was significantly greater among pets from COVID-19-positive households compared to those with owners of unknown status. In the Italian study they found 12.8% (6/47) of dogs with anti-SARS-CoV-2 canine immunoglobulins in COVID-19-positive households, but only 1.5% (2/133) of dogs living in COVID-19-negative households. Of note, in our study the influence of the family environment was not evaluated in the group of sick dogs. Some owners presented COVID-19 symptoms, nevertheless they were not tested for SARS-CoV-2. Other owners had not presented symptoms during the pandemic. Therefore, it was decided to exclude this data due to a lack of reliable information.

Most people infected with SARS-CoV-2 display an antibody response between day 10 and day 21 after infection, and several studies have suggested that previous antibodies and T cells against endemic human coronavirus may provide some degree of cross-protection from SARS-CoV-2 infection [35]. Further, there have been reports of pre-existing memory CD4 + T cells that are cross-reactive with SARS-CoV-2 and the common human cold coronaviruses HCoV-OC43, HCoV-229E, HCoV-NL63, or HCoV-HKU1 [36]. In our data, we did not find any correlation between α-CCoV and α-SARS-CoV-2 IgG-positivity, although the low number of cases makes it difficult to reach a valid conclusion. Some viruses as CAV, CPV and CDV cause pathologies affecting puppies with high mortality rates if they are not treated. Most routine protocols include the vaccination against these viruses, even before the pup is allowed to be in contact with other dogs. Since most of the dogs are vaccinated to this pathology we rule out any correlation with anti-SARS-CoV-2 in our group of study.

In summary, we analyzed dogs affected by severe pulmonary disease, all of which were negative for SARS-CoV-2 by RT-qPCR. However, one sick dog (1/17) and five healthy dogs living in COVID-19-positive households (5/20) presented α-SARS-CoV-2 IgG. Our results suggest that even in the case of a canine infection it would be poorly transmissible. Moreover, dogs with owners positive for SARS-CoV-2 could have been more likely to be exposed to infection during outbreaks.

## Supplementary Information

**Additional file 1.** Vaccination status from sick and healthy dogs.

**Additional file 2.** Immune response of sick and healthy dogs.

## Abbreviations

a.u.: Arbitrary units
CaHV-1: Canine herpesvirus-1
CAV: Canine adenovirus
CCoV: Canine enteric coronavirus
CDV: Canine distemper virus
CIV: Canine influenza A virus
CoV: Coronaviruses
COVID-19: Coronavirus disease 2019
CPIV: Canine parainfluenza virus
CPV: Canine parvovirus
CRCoV: Canine respiratory coronavirus
CT: Computed tomographic
CXR: Chest X-ray
DV: Dorso-Ventral
HRP: Horse radish peroxidase
IgG: Immunoglobulins
IQR: Interquartile range
LL: Latero-Lateral
RT-qPCR: Reverse transcription quantitative PCR
SARS: Severe acute respiratory syndrome
SD: Standard deviation
WBC: White blood cells
α-CoV: Alphacoronavirus
β-CoV: Betacoronavirus
γ-CoV: Gammacoronavirus
δ-CoV: Deltacoronavirus
