# Supplementary material for: Humoral responses to SARS-CoV-2 by healthy and sick dogs during the COVID-19 pandemic in Spain
Source: Vet Res. 2021 Feb 15;52:22. doi: 10.1186/s13567-021-00897-y (PMC7883760; doi:10.1186/s13567-021-00897-y)
Supplement: Supplementary file 1 — Additional file 1. Vaccination status from sick and healthy dogs. [file 13567_2021_897_MOESM1_ESM.docx]

**Additional file 1 Vaccination status from sick and healthy dogs.**

|  | | **CCoV** | **ICH** | **CPV** | **CDV** | ***Bordetella*** | **CPIV** | ***Leptospira*** |
| --- | --- | --- | --- | --- | --- | --- | --- | --- |
| **Sick dogs** | **SER 01** | no | no | no | no | no | no | no |
|  | **SER 02** | yes | yes | yes | yes | yes | yes | yes |
|  | **SER 03** | yes | yes | yes | yes | yes | yes | yes |
|  | **SER 04** | yes | yes | yes | yes | yes | yes | yes |
|  | **SER 05** | yes | yes | yes | yes | yes | yes | yes |
|  | **SER 06** | no | no | no | no | no | no | no |
|  | **SER 07** | no | no | no | no | no | no | no |
|  | **SER 08** | yes | yes | yes | yes | yes | yes | yes |
|  | **SER 09** | yes | yes | yes | yes | yes | yes | yes |
|  | **SER 10** | yes | yes | yes | yes | yes | yes | yes |
|  | **SER 11** | no | no | no | no | no | no | no |
|  | **SER 12** | yes | yes | yes | yes | yes | yes | yes |
|  | **SER 13** | yes | yes | yes | yes | yes | yes | yes |
|  | **SER 14** | no | no | no | no | no | no | no |
|  | **SER 15** | no | no | no | no | no | no | no |
|  | **SER 16** | no | no | no | no | no | no | no |
|  | **SER 17** | yes | yes | yes | yes | yes | yes | yes |
|  | **SER 18** | no | no | no | no | no | no | no |
| **Healthy dogs** | **SER 101** | no | yes | yes | yes | yes | no | yes |
|  | **SER 102** | no | yes | yes | yes | yes | no | yes |
|  | **SER 103** | no | yes | yes | yes | yes | yes | yes |
|  | **SER 104** | yes | yes | yes | yes | yes | yes | yes |
|  | **SER 105** | no | yes | yes | yes | yes | no | no |
|  | **SER 106** | yes | yes | yes | yes | dk/na | yes | yes |
|  | **SER 107** | no | yes | yes | yes | yes | yes | yes |
|  | **SER 108** | dk/na | yes | yes | yes | dk/na | dk/na | dk/na |
|  | **SER 109** | dk/na | yes | yes | yes | dk/na | dk/na | dk/na |
|  | **SER 110** | no | yes | yes | yes | yes | yes | yes |
|  | **SER 111** | no | yes | yes | yes | yes | yes | yes |
|  | **SER 112** | no | yes | yes | yes | yes | yes | yes |
|  | **SER 113** | no | yes | yes | yes | no | no | yes |
|  | **SER 114** | dk/na | yes | yes | yes | yes | yes | yes |
|  | **SER 115** | yes | yes | yes | yes | no | yes | yes |
|  | **SER 116** | yes | yes | yes | yes | no | yes | yes |
|  | **SER 117** | no | yes | yes | yes | yes | no | no |
|  | **SER 118** | yes | yes | yes | yes | yes | yes | yes |
|  | **SER 119** | dk/na | yes | yes | yes | yes | dk/na | yes |
|  | **SER 120** | dk/na | yes | yes | yes | yes | dk/na | yes |

Vaccines administered (yes, gray box); not administered (no, white box); don’t know/no answer (dk/na). CCoV: canine coronavirus; ICH: infectious canine hepatitis; CPV: canine parvovirus; CDV: canine distemper virus; CPIV: canine parainfluenza virus.
