# Supplementary material for: Humoral responses to SARS-CoV-2 by healthy and sick dogs during the COVID-19 pandemic in Spain
Source: Vet Res. 2021 Feb 15;52:22. doi: 10.1186/s13567-021-00897-y (PMC7883760; doi:10.1186/s13567-021-00897-y)
Supplement: Supplementary file 2 — Additional file 2. Immune response of sick and healthy dogs. [file 13567_2021_897_MOESM2_ESM.docx]

**Additional file 2 Immune response of sick and healthy dogs.**

|  | | **α-CAV** | **α-CPV** | **α-CDV** | **α-CCoV** | **α-CCoV** | **α-SARS-CoV-2** |
| --- | --- | --- | --- | --- | --- | --- | --- |
|  |  | **IgG** | **IgG** | **IgG** | **IgM** | **IgG** | **IgG** |
| **Sick dogs** | **SER 01** | + | + | + | + | + | - |
|  | **SER 02** | + | + | + | - | + | - |
|  | **SER 03** | nd | nd | nd | nd | nd | nd |
|  | **SER 04** | + | - | + | - | - | - |
|  | **SER 05** | + | + | + | - | + | - |
|  | **SER 06** | + | + | + | - | - | - |
|  | **SER 07** | + | + | + | - | - | - |
|  | **SER 08** | + | + | - | - | - | - |
|  | **SER 09** | + | + | + | + | + | - |
|  | **SER 10** | + | + | + | - | + | - |
|  | **SER 11** | - | + | - | - | - | - |
|  | **SER 12** | + | + | + | - | - | - |
|  | **SER 13** | + | + | + | - | - | - |
|  | **SER 14** | + | + | + | + | - | - |
|  | **SER 15** | - | + | + | - | - | + |
|  | **SER 16** | + | + | + | - | - | - |
|  | **SER 17** | + | + | + | - | + | - |
|  | **SER 18** | + | + | + | - | + | - |
| **Healthy dogs** | **SER 101** | + | + | + | - | - | + |
|  | **SER 102** | + | + | + | - | - | + |
|  | **SER 103** | + | + | + | - | - | - |
|  | **SER 104** | + | + | - | - | + | - |
|  | **SER 105** | + | + | + | - | + | - |
|  | **SER 106** | - | - | + | - | + | - |
|  | **SER 107** | + | + | + | - | - | - |
|  | **SER 108** | + | + | - | - | + | - |
|  | **SER 109** | - | + | - | - | - | - |
|  | **SER 110** | + | + | + | - | + | + |
|  | **SER 111** | + | + | + | - | + | - |
|  | **SER 112** | + | + | + | - | + | - |
|  | **SER 113** | + | + | + | - | + | - |
|  | **SER 114** | + | + | + | - | + | - |
|  | **SER 115** | + | + | + | - | - | - |
|  | **SER 116** | + | + | + | - | + | - |
|  | **SER 117** | + | + | + | - | + | + |
|  | **SER 118** | + | + | + | - | + | - |
|  | **SER 119** | + | + | + | + | - | - |
|  | **SER 120** | + | + | + | - | - | + |

CAV: canine adenovirus; CPV: canine parvovirus; CDV: canine distemper virus; CCoV: canine coronavirus; SARS-CoV-2: severe acute respiratory syndrome coronavirus 2; IgG: immunoglobulin G; IgM: immunoglobulin M. Presence (+), absence (-), not determined (nd).
